# Complete Genome Sequence of the Goatpox Virus Strain Gorgan Obtained Directly from a Commercial Live Attenuated Vaccine

**DOI:** 10.1128/genomeA.01113-16

**Published:** 2016-10-13

**Authors:** Elisabeth Mathijs, Frank Vandenbussche, Andy Haegeman, Ahmad Al-Majali, Kris De Clercq, Steven Van Borm

**Affiliations:** a Molecular Platform, Veterinary and Agrochemical Research Centre, Ukkel, Belgium; b Viral Diseases, Vesicular and Exotic Diseases, Veterinary and Agrochemical Research Centre, Ukkel, Belgium; c Jordan Bio-Industries Centre (JOVAC), Amman, Jordan; d Faculty of Veterinary Medicine, Jordan University of Science and Technology, Irbid, Jordan

## Abstract

This is a report of the complete genome sequence of the goatpox virus strain Gorgan, which was obtained directly from a commercial live attenuated vaccine (Caprivac, Jordan Bio-Industries Centre).

## GENOME ANNOUNCEMENT

Capripox disease is an economically important disease in small domestic ruminants that is caused by goatpox virus (GTPV) and sheeppox virus (SPPV), which belong to the *Poxviridae* family, *Capripoxvirus* genus. The disease is present in central and north Africa, the Middle East, and various parts of Asia, with occasional incursions in southern Europe. Vaccination plays a key role in controlling the spread of the disease and is mainly based upon the use of live attenuated vaccines. The genomic characterization of the virus strains that compose these vaccines, along with *in vivo* experimentations, will give insight into the mechanism of attenuation for vaccine strains and will improve our understanding of vaccination failure observed in the field (1). Here, we report the complete genome sequence of the GTPV strain Gorgan, obtained directly from a commercial live attenuated vaccine (Caprivac, Jordan Bio-Industries Centre).

DNA was purified from a freeze-dried vaccine pellet dissolved in 3 ml of phosphate-buffered saline using the Puregene Core Kit A (Qiagen) according to the manufacturer’s instructions. Presequencing enrichment was performed through an in-house long-range PCR methodology covering the entire genome with overlapping ~5.5-kb amplicons. All amplicons were pooled in an equimolar manner. P6-C4 sequencing was performed on a single-molecule real-time (SMRT) cell on a PacBio RSII sequencer (Pacific Biosciences) at the Genomics Core UZ Leuven (Leuven, Belgium).

Consensus amplicon sequences were obtained from the reads using the Long_Amplicon_Analysis protocol (default parameters; Pacific Biosciences) in SMRT Portal version 2.3.0 (Pacific Biosciences). The amplicons were further assembled using iAssembler software version 1.3.2 (2). Discrepancies with previously published GTPV genomes were confirmed by Sanger sequencing. The protein-coding genes were predicted by NCBI’s ORF-Finder (http://www.ncbi.nlm.nih.gov/orffinder) and by GATU relative to GTPV Pellor reference sequence NC_003027 (3).

Consensus amplicon sequences were assembled into a single double-stranded, linear DNA sequence of 148,146 bp, with an average G+C content of 25.33%. The GTPV strain Gorgan obtained directly from the Caprivac vaccine contains a 143,732-bp central coding region flanked by two identical terminal-inverted repeats of 2,144 bp. In comparison with GTPV strain Pellor, the Gorgan strain genome is characterized by two single-nucleotide substitutions (N/D in GTPV_gp34 and N/D in GTPV_gp63) and a large deletion of 1,593 bp disrupting two putative genes (GTPV_gp137 and GTPV_gp138).

### Accession number(s).

The complete genome sequence of GTPV strain Gorgan (Caprivac vaccine) has been deposited in GenBank under the accession number KX576657.
